# The Certification and Registration of Deaths

**Published:** 1909-03-20

**Authors:** 


### SPECIAL ARTICLE.

### THE CERTIFICATION AND REGISTRATION OF DEATHS.

As is well known, medical practitioners are called upon to certify deaths so frequently that the certification of deaths may be said to be one of their daily duties; and the registrar copies into his books the cause or causes of death, etc., as certified by the medical practitioner. It is also common knowledge that at short intervals, generally one week, the registrars send a complete list of deaths containing information as to date of death, locality, name and surname, sex, age, rank or profession, medical attendant, and, lastly, but of the greatest importance, the cause of death, to the medical officer of health of the district concerned.

The medical officer of health, therefore, is dependent upon the general practitioner, to a great extent, for the accuracy or otherwise of his mortality statistics. The importance of this is obvious when it is remembered that such statistics are the foundation of sanitary work, and that they allow comparisons to be made over a series of years which show the actual results of many sanitary reforms. The part which the general practitioner has played already in facilitating these comparisons, although they are not free from error, is of no small moment. For example, if it should be desired at any time to indicate the progress or otherwise which a borough or district has made during a series of years in the mortality from infectious diseases, phthisis, cancer, etc., the number of deaths as certified must be ascertained. From these figures the death rates from any given cause are calculated per 1,000 of the population living. In the same way we may ascertain the geographical distribution of fatal diseases, and the relative incidence of mortality associated with various trades and occupations, different age periods, the sexes, and different social classes.

Any system of certification and registration of deaths should not only result in accuracy of statistics, but should be effective in preventing crime. It does not seem satisfactory that a body can be buried without full medical certification as to the cause of death. Under the present conditions it is unlikely that mortality statistics can be absolutely correct; but many improvements might be made which would ensure greater accuracy, and which would give greater satisfaction alike to the general practitioner and the health official.

### Certified Deaths.

The Registration Act of 1874 made it compulsory on medical men to give a certificate stating the cause of death to the best of their knowledge and belief of any patients who had been attended by them. There is no obligation upon the medical man to ascertain that the patient is actually dead. Some defects in medical death certificates may now be mentioned. Occasionally there is too much information on the death certificate—*e.g.* the following diseases have appeared on the same certificate: apoplexy, diarrhœa, and bronchitis. On the other hand, the death certificate may contain insufficient information—*e.g.* the word " catarrh " has been given as the cause of death without indication as to the organ affected. Again, the word " dropsy " has been stated to be the cause of death without any reference to heart, kidneys, or liver. Generally speaking, it would be better if all words indicating symptoms were avoided on death certificates in stating the primary cause of death. The writer is well aware of the difficulty in always avoiding the use of such terms as inanition, marasmus, senile decay, etc.; but Sir Robert Christison used to state that death from natural decay was little more than an admission of ignorance, and that even the oldest died from some definite and ascertainable organic lesion. This statement may not be true in every case, but in many it is so.

Certificates which give such names as jaundice or hæmorrhage as the primary cause of death are of little use for purposes of classification. As is well known, jaundice may indicate malignant disease, gall-stones, or catarrh of the bile ducts; and hæmorrhage may indicate lesions of the lungs, stomach, intestines, uterus, and blood vessels, or may be associated with a traumatic origin. Septicæmia given alone as the cause of death is unsatisfactory; for it may be associated with the puerperal state or traumatic causes. It may be well to state here that the term puerperal fever has been removed from the nomenclature of the Royal College of Physicians. Pyæmia, septicæmia, or septic intoxication occurring in puerperal women should be described as puerperal pyæmia, puerperal septicæmia, or puerperal septic intoxication.

There is no doubt that in certain cases the exact cause of death is not given even when it is known. In this connection reference may be made to such causes of death as alcoholism and syphilis. There is, perhaps, some excuse for this, owing to the fact that death certificates written by medical men are not confidential documents, and that copies of the same are made by registrars of births and deaths for submission to various life insurance companies. Another source of error which prevents an accurate classification of death is that sometimes a secondary cause of death is given as the primary cause. Care should be taken whenever possible to record causes of death in their chronological order. Here, also, it may be stated that the Registrar-General has not issued any detailed instructions as to what is meant by the last illness. This may give rise to some confusion.

Another class of deaths to which reference should be made are those which are certified after an inquest has been held by a coroner. As is well known, coroners often dispense with both medical evidence and post-mortem examinations, and accept the verdict of the " twelve men good and true." Owing to the fact that the majority of coroners have no medical knowledge, and that they lead juries who also have no medical knowledge, this is very unfortunate from the point of view of an accurate classification of deaths. The system in certain localities of appointing coroners from the ranks of the medical profession is certainly one to be encouraged. It is recorded that at an inquest in a Yorkshire town within the last two or three years the verdict was as follows: " Death was due to strangulation, but there was no evidence to show that the child had ever lived." And frequently the result of the inquest is the verdict " Death from natural causes," when it is likely that a more correct cause of death would have been ascertained after efficient medical investigation.

### Uncertified Deaths.

Those deaths which are registered without the certificate of a registered general practitioner, or without the certificate of a coroner after an inquest, are classed among the uncertified deaths. Although the proportion of uncertified deaths is not so large as it was several years ago, there is still great need for improvement in this respect. Some of these deaths probably have been mentioned to the coroner and some inquiry made by a lay person. Again, the registrar can accept the certificate of unqualified people. Also many of these deaths refer to children under the age of one year. At present there is no compulsory official registration of still births, and it certainly seems anomalous that a midwife can issue a burial certificate for a still-born child. In this respect the Registration Act needs amendment.

A Select Committee was appointed in 1893 to inquire into this subject. They issued a report containing the following important conclusions and recommendations:

1. That in no case should a death be registered unless on the certificate of a registered medical practioner or of the coroner after inquest.

2. That in each sanitary district a public medical certifier should be appointed to investigate all deaths uncertified, as tried at Edinburgh and Manchester.

3. That the fact of death should be verified by the medical attendant, but if on the ground of distance or other sufficient reason, then a certificate signed by two persons verifying the death.

4. That medical men should be required to send certificates of the cause of death direct to the registrar.

5. That a compulsory form of certificate should be prescribed.

6. That still-births which have reached the stage of development of seven months should require registration upon the certificate of a registered medical practitioner.

7. That burial should take place only on the certificate of the registrar.

It also seems desirable that all medical death certificates should be paid for by the national exchequer, and that they should be sent by medical practitioners direct and under sealed cover to the registrar. This would probably result in a more accurate statement of the cause of death, and the medical man would feel that he was being paid for skilled work, for which at present he receives no payment.

It would also be desirable that the Registrar-General should instruct registrars not to copy in full the cause of death as given in the medical certificates for insurance societies. If these societies required the information they should obtain it at the discretion of the Registrar-General. If a man is insured who believes himself well, and in whom the medical examiner of the insurance company can find no evidence of disease, his relatives should receive the benefit of such insurance. Such reforms would ensure greater accuracy of death returns and statistics, and the abolition of uncertified deaths would tend to diminish neglect of young children and old persons, to lessen a certain amount of carelessness during pregnancy, and to reduce the death rate amongst illegitimate children.

